# The Impact of “Wine Country of Origin” on the Perception of Wines by South African and French Wine Consumers: A Cross-Cultural Comparison

**DOI:** 10.3390/foods10081710

**Published:** 2021-07-23

**Authors:** Dominique Valentin, Carlo Valente, Jordi Ballester, Ronan Symoneaux, Ina Smith, Florian F. Bauer, Helene Nieuwoudt

**Affiliations:** 1 Centre des Sciences du Goût et de l’Alimentation, Université Bourgogne Franche-Comté, AgroSup Dijon, CNRS, INRA, F-21000 Dijon, France; jordi.ballester@u-bourgogne.fr; 2 South African Grape and Wine Research Institute, Department of Viticulture and Oenology, Stellenbosch University, Stellenbosch 7600, South Africa; carlo_valente@bat.com (C.V.); fb2@sun.ac.za (F.F.B.); hhn@sun.ac.za (H.N.); 3 Groupe ESA, UPSP GRAPPE, Ecole Supérieure d’Agricultures, SFR 4207 QUASAV, 55 Rue Rabelais, BP 30748, 49007 Angers, France; r.symoneaux@groupe-esa.com; 4 Chenin Blanc Association, Stellenbosch 7600, South Africa; ina.smith@iafrica.com

**Keywords:** consumers, Chenin wine, country of origin, representation, categorization

## Abstract

Culture is an important factor that influences how marketing interacts with food choice. This study aims at exploring the effect of consumers’ Country of Origin (COO) on wine representations and perception using Chenin blanc as a model. The first objective was to evaluate the role of origin in the construction of the representation. We used the theoretical framework of social representation to compare South African (SA) and French consumers’ representations via a word association task. The results indicated that SA representations are dominated by consumers’ experience of the wine (sensory and emotional dimensions), whereas French representations are dominated by the wine itself, in particular its origin and mode of consumption. The second objective was to evaluate the effect of origin on wine categorization in two conditions: with and without information concerning the two geographical origins of the samples. Results showed that providing information on the origin of the wines affected French participants more than SA participants. In both conditions, the groups of wines formed in the sorting tasks by SA participants were based on sensory descriptors and appeared not to be impacted by the information on origin. In contrast, providing information on the origin of the wines to French participants led to an increased use of the words “Loire”, “South Africa” and “familiar” suggesting a different sorting strategy more deliberately based on the origin of the wines. Our findings have important implications for the marketing and export activities within the wine industry.

## 1. Introduction

For products sold into international markets, understanding how consumers’ culture interact with marketing strategies is a crucial aspect of predicting products’ success. To buy a bottle of wine, consumers around the world are faced with a broad choice of wine brands. In this context, the origin of the wine has become increasingly more important in purchase decisions. Lacey and coworkers [1], for example, found that, for Australian consumers in a fine restaurant setting, regionality and grape variety were the strongest attributes taken into consideration when selecting a wine to go with their meal. Ginon and coworkers [2] found that region of origin was the second most important choice criteria for Burgundy consumers. A study by Atkin & Johnson [3] showed that brand and place-of-origin information such as region, country and state were the most important attributes in the consumers’ choice of wine.

Consumers use Country Of Origin (COO) as a “halo” from which to infer product attributes (see [4] for a review on COO effects), thus reducing the perceived risk in the decision making process of purchasing wine. Knowing the origin of wine has also been shown to affect consumers’ perception of wines. For example, a study by Ashton [5] found that when a wine is believed to be from New Jersey, it receives “lower enjoyment ratings’’ than when the identical wine is believed to be from California. Veale and Quester [6] also showed that COO can change how consumers perceive and evaluate wine. In their study, the sensory quality of wines was reduced by increasing the sourness and astringency. The impact was masked when wines were labelled with information on COO and price. A recent study [7] showed that the COO of wines affects more wine-traders’ representation of wines than wine-traders’ own COO. This effect of COO, however, can change overtime, or with wine educational marketing initiative [8].

In this article, we explore the effect of consumer and wine COO on wine representations. Contrary to previous studies dealing with wine in general, we focused on a specific white cultivar: Chenin blanc. Chenin blanc has its origin in France from where it has spread and is nowadays planted across the globe. Statistics for 2016 reported that some 32,000 hectares are under Chenin blanc plantings in 22 different countries [9]. Four countries account for 97% of the plantings, namely South Africa (SA) 55%, France 29%, Argentina 7% and the United States of America 6%. Research suggests that the cultivar is interesting from viticultural, winemaking and sensory perspectives. The aroma of Chenin blanc grapes has been described as neutral [10] since it lacks some dominant distinct flavours such as those associated with the aromatic white cultivars Weisser Riesling, Gewürtztraminer, Sauvignon blanc and Muscat d’Alexandrie, amongst others [11,12]. Nevertheless, Chenin blanc wines have fairly recognizable, albeit diverse sensory profiles associated with the cultivar [13,14,15]. Concerning the taste, Chenin blanc is characterized by high acidity [16]. Research has also shown that the wines produced from Chenin blanc vineyards gave good expressions of the terroir (soil type, climate) in specific locations in the Loire Valley, France [17]. Dry and sweet wines are produced and flavors can be manipulated during winemaking through yeast selection [18,19,20,21], skin contact [22] and wood contact [23]. As such, Chenin blanc wines constitute a good model for looking at the effect of consumers’ COO on wine representations.

With SA and France being the largest producers of Chenin blanc wine, we focused our study on consumers from these two countries. SA is the largest Chenin blanc wine-producing country in the world, with Chenin blanc, also known as Steen, reportedly one of the earliest grapes to be cultivated at the Cape of Good Hope by the Dutch colonists in the 17th century [24,25]. Nowadays, Chenin blanc is an economically important white wine cultivar in SA; it shows good adaptation to the changing climate and resulting drought conditions and is cultivated in hot inland regions, as well as cooler climate areas [26,27]. In the past, the cultivar was not considered as supporting quality wine production due to it being primarily used for the production of bulk wines. However, over the last two decades, significant efforts have been made to improve viticultural and oenological practices, and to produce high-quality Chenin blanc wines that reflect the character of the varietal and the geographical origin. Results from tastings and wine competitions suggest that this effort has been by-and-large successful, and the figures of locally consumed and export volumes have shown steady increases [27].

France is a historic wine producer and the symbol of old-world wine. Even though consumption decreased over the last 40 years, wine remains an expression of national identity and a way of regional differentiation. Chenin blanc is native to France and some authors have mentioned that its name appeared between the 10th and 11th century [16]. Today, Chenin blanc is planted on 1.2% of the French vineyard, but it is specifically produced in the Loire Valley. In 2016, 95% of the 9500 ha planted in Chenin were in this region. This white grape is used in nearly 12 dry Protected Designations of Origin (PDO) wines of the Loire Valley, including Anjou, Saumur, Vouvray and Savennieres, eight sweet PDO wines and four sparkling PDO wines making this the best-known grape variety in the region [28].

Our objective was twofold. First, we were interested in investigating what concepts consumers from FR and SA associate with dry Chenin blanc wine and, in particular, whether they would spontaneously link Chenin blanc sec to its origin. We used the theoretical framework of social representation (i.e., opinions, knowledge, and beliefs that result from a social construction of reality [29]), in particular the prototypical structural approach (or central core theory [30]), to explore SA and French consumer representations of Chenin blanc wine. This approach provides access to the content and organization of the representations by asking participants to list the words or expressions that come to their mind in reference to the object under study (free association task, see [31] for a recent review) and to rate their importance and valence. It relies on the postulate that a social representation is a hierarchized and organized system, composed of two interactive sub-systems: a central system and a peripheral system. The core system constitutes the common and consensual base of the representation, and the peripheral system gives concrete expression and flexibility to the representation.

Our second objective was to evaluate whether providing information on the origin of the wine (France vs. SA) would modify the way participants would perceive and categorize the wines. Categorization is a cognitive process whose goal is to provide maximum information with the least cognitive effort. It leads to a simplified version of the world. Looking at how participants categorize objects provides an indication of the salience of the dimensions underlying their thought processes. Dimensions of the object are important to guide their decisions. Free sorting tasks [32] are an easy way to access these dimensions and to evaluate whether they can be modulated by external variables. Participants had to sort a set of French and SA Chenin blanc wines either in a blind condition or in an informed condition in which they were told that the wines came from France or SA.

Gaining knowledge into the concepts associated with Chenin blanc wines and understanding the effect of origin on their categorization might provide practical insights into adapting both national and international marketing strategies. Our hypothesis is that consumers’ representation of Chenin blanc depends on their experience with wine and the cultural context surrounding wine consumption.

## 2. Materials and Methods

### 2.1. Wines

Fourteen wines were selected for the study: seven from France and seven from SA (Table 1). All wines were 100% Chenin blanc variety. The price ranges per bottle were 12–24 € (average = 16.85) for French wines and 4–9 € (average = 7.5) for SA wines.

The selection of the wines was done in a blind tasting in SA, by a panel of 10 people that consisted of five Chenin blanc industry experts and five members (staff and post-graduate students) of the Department of Viticulture and Oenology, Stellenbosch University, SA, who were actively involved with sensory research on Chenin blanc wine. The industry experts were well acquainted with the Chenin blanc wines from both countries. The Chenin blanc producers on the panel have visited the Loire several times and also gained detailed practical experience of Chenin blanc production in France during several harvests. The Loire list of samples included wines from each of the most important PDOs. (Anjou, Vouvray, Savennières and Saumur). It was also taken into account that the residual sugar (RS) of the wines selected were all 5 g/L or less (SA regulations for dry wines are ≤5 g/L RS). The vintages of the selected wines ranged from 2010 to 2013.

### 2.2. Participants

A total of 99 Chenin blanc consumers was recruited in France and 107 in SA. In France, participants were recruited in Angers and in SA in Stellenbosch, two Chenin blanc growing areas. A frequency of consumption questionnaire was used to select consumers drinking white wines at least a few times a month and who declared consuming Chenin blanc sec (Vouvray sec, Anjou sec, Saumur sec or Savennières sec in France). The demographics of the participants are presented in Table 2. The SA panels were younger on average and included more women than the French panels. This is in keeping with white wine consumption patterns in SA, which showed consistent above-average consumption by females in the 20 to 40 years age segment of medium-priced wines such as those used in this study [33]. None of the participants was a wine professional. Informed written consent was obtained from each participant prior to participation.

### 2.3. Procedure

The procedure included three steps: a word association task, a sorting task, and a habit and sociodemographic questionnaire. Participants were seated at separate tables and provided with basic information about the study. They were advised that they would taste and make judgments about 14 wines and that all wines were Chenin blanc sec. They were advised that all the wines had to be expectorated (i.e., not swallowed). All participants started with the word association task followed by the sorting task. The task was performed in the official language of the respective countries, namely in French in France and in English in SA. All tasters in SA were fluent in English.

#### 2.3.1. Word Association Task

Participants were given stimulus words or phrases and, for each word or phrase they were asked: 1) to write down the first four words that came to their mind, 2) to rate the importance of each word on a seven-point scale anchored “not important” on the left and “very important” on the right, and 3) to rate the valence of each word on a seven-point scale anchored “very negative” on the left and “very positive” on the right. Each participant received a six-page booklet containing the instructions on the first page and three stimulus words on the following three pages. The first two stimulus words (“sky” and “hammer”) served as warm up words to familiarize the participants with the task. The third stimulus phrase was the target phrase “Chenin blanc sec”. The fifth and sixth pages contained the scales to score the importance and valence of the four words generated by the participants for the target phrase “Chenin blanc sec”.

#### 2.3.2. Sorting Task

Participants received the 14 wines presented in a different order for each participant according to a Williams latin square arrangement generated by FIZZ software (Biosystemes, Courtenon, France). They were first required to smell and taste each wine once in the proposed order. Then, they had to sort the wines according to their similarity. Participants were not bound by time constraints and were allowed to sort the wines in as many groups as they felt necessary. Thereafter, they could re-evaluate the wines as many times as they wanted and in any order. After they finished their sorting task, participants were asked to describe each group of wines with some words. Half of the participants did not receive information on the origin of the wines (no information condition). The other half were told that the wines had two origins: SA and Loire in France (information condition).

### 2.4. Experimental Conditions

In both countries, the tests were conducted in a lecture room equipped with individual tables spaced to avoid communication between panellists, in a single session lasting approximately one hour. The wines were kept between 8–12 °C and taken out of the storage cabinet 30 min before serving. A new bottle of each wine was opened daily throughout the duration of the experiment and were first checked for faults. Forty mL samples were poured into standardized black tasting glasses to eliminate visual cues as sources of information. The glasses were coded with 3-digit numbers and were covered with odourless plastic Petri dishes. Water and crackers were available throughout the session for palate rinsing purpose.

### 2.5. Data Analysis

#### 2.5.1. Word Association Task

##### Textual Analysis

All analyses were carried out on the original languages (i.e., French in France and English in SA). A lemmatization was first carried out in the original language on the FR and SA corpus by which the terms were reduced to their simplest form. This was done by native speakers in each country.

A diversity and a rarity index were computed to compare the corpora obtained in France and SA. The diversity index is computed as the ratio of the number of different evoked words to the total number of evoked words. The larger the index, the larger the diversity (a value of 1 indicates a maximum diversity). The rarity index corresponds to the number of hapaxes (i.e., words cited only once) divided by the number of different evoked words [34].

Then the lemmatized terms were grouped into general categories. The categorization was done in the original language by one bilingual researcher following the same grouping rules for the two corpora. The categorizations were then checked by independent researchers in the two countries. After a discussion undertaken to obtain a consensus regarding the generated categories, a common label was assigned to each of the definitive categories.

Frequencies of elicitation were obtained by counting the number of participants who elicited the words after lemmatization in each category. Chi-square tests were conducted to examine differences in the use of categories between countries. The average importance and valence were also computed after lemmatization in each category. One way Analyses of variance (ANOVA) were computed to test the effect of Consumers’ COO on category importance and valence.

##### Prototypical Analysis

A prototypical analysis of the evoked terms was used to define the hierarchical structure of the Chenin blanc wine representations in FR and SA. This analysis highlights the salience of certain elements of the representation by crossing the frequency of occurrence and the importance of these elements. A frequency and an importance cutting point are calculated to separate the elements of the representation in a central zone and three peripheral zones.

The analysis was performed after lemmatization. Only words with a frequency of citation greater than two were kept. Two indices were first computed: the frequency of citation of the evoked terms, which indicates the degree of words-sharing among participants, and the average importance of the words, which is an indicator of the saliency of the evoked words. Then a frequency and an importance cut-off point were determined. The frequency cut-off point was obtained by visually displaying the frequency of occurrence of words in decreasing order [34]. The cut-off point was taken to be the frequency at which the difference between two successive frequencies is maximal. For comparison purposes, the same frequency cut-off was used for the two groups of consumers. The words with a frequency higher than the frequency cut-off point were classified as “high frequency” and the other ones as “low frequency”. The importance cut-off point was obtained by averaging the importance criteria across categories [35]. The words with importance criteria higher than the importance cut-off point were classified as high importance and the other ones as low importance. The words were then cross-tabulated in a 2 × 2 table representing the central core of the representation (words with high frequency and high importance) and the peripheral elements. The words in the central core zone are stable and consensual, shared by all, and they provide a framework for interpreting and categorizing new information. The peripheral elements support the heterogeneity of the group. They are flexible and allow the adaptation to reality. They are organized into three categories: The first periphery zone includes the responses with high frequency and low importance. They are salient responses that, however, indicate secondary elements of the representation. The second periphery or contrast zone includes low frequency of elicitation (less shared elements), but are considered as being very important. This zone indicates the existence of a subgroup that consistently values some elements differently from the majority. The third periphery corresponds to words with low frequency and low importance. This periphery allows space for more individual ideas.

#### 2.5.2. Sorting Task

The sorting results of each participant were encoded in individual *wine* * *wine* distance matrices where the rows and the columns are wines. A value of 0 between a row and a column indicates that the participant put both wines together, and a value of 1 indicates that the wines were not put in the same group.

A global analysis was first carried out to compare the four conditions (French without information, French with information, SA without information and SA with information). Individual distance matrices were summed for each condition. The four resulting distance matrices were submitted to a 3-way multidimensional scaling analysis (Distatis, [35]). This analysis gives rise to a RV map representing the relative distances between the original matrices (RV coefficients are multivariate generalization of the squared Pearson correlation coefficient). It was used to compare the sorts obtained in the four conditions. The closer the conditions on the map the more similar the sorts performed by the participants.

Then separate Distatis analyses were carried out on individual data to analyze the distances between wines in each condition. These analyses produce compromise maps of the positioning of the wines in each condition. Two wines close together on these maps were often sorted together and two wines far away were rarely sorted together. A bootstrap resampling technique with replacements was used to estimate the reliability of the position of the products in the compromise map. A confidence interval around each wine was calculated at a 95% tolerance interval [36]. The descriptors associated with the groups of products were first lemmatized and categorized using SPAD 9.3 Text mining procedure (Coheris). Then a wine*descriptor contingency table was built by condition and projected as supplementary variables on the Distatis compromise space.

All Distatis Analyses were carried out with The R Package DistatisR (available from https://github.com/HerveAbdi/DistatisR, accessed on 20 July 2021).

## 3. Results

### 3.1. Word Association Task

To evaluate the role of origin in constructing the representation of Chenin blanc, we analyzed the results of the word association task. This task was carried out before the sorting task.

#### 3.1.1. Textual Analysis

A total of 500 words were recorded in France and 535 in SA before lemmatization. Among those words, 154 and 180 distinct words were obtained in France and SA after lemmatization. The relatively low diversity (0.31 for FR and 0.34 for SA) and rarity (0.18 for FR and 0.19) indexes computed for the French and SA corpora suggest the existence of a structured representation for both groups. The words generated by French and SA participants were classified into eight categories (Table 3): Consumption mode (e.g., friends, aperitif, summer, meals), Emotional (e.g., pleasant, nice, joy), Food (e.g., fish, shellfish), Origin (e.g., Anjou, Loire, terroir, SA), Sensorial (e.g., acidic, bitter, fruity), Usage (e.g., drink, bottle), Viticulture/enology (e.g., vines, variety, grape), and Other (e.g., alcohol). A global chi-square analysis showed an effect of consumers’ COO on the saliency of these categories (chi-square = 207.376, *p* < 0.0001).

The SA corpus is dominated by the sensory dimension at 59% compared to 21% in the French corpus. The diversity of the terms elicited by more than two participants in the sensorial category also reflects the saliency of this dimension in the representation of Chenin blanc wine in SA (35 against 12 in France). The sensory words generated in SA are more specific than the words generated in France, especially in terms of aromas (19 terms against 2). The words more often used in this category were *taste*, *fruit*, *tasting*, *color*, *aroma*, *white* for France and *fruity*, *wood*, *acidic*, *crisp*, *white*, *tropical* for SA.

French participants elicited significantly more words from the categories viticulture/enology (29 vs. 12%), origin (15 vs. 3.7%), food (8.6 vs. 3.2%) and usage (4.2 vs. 1.5%) than SA participants. In these categories, the more frequently used words were *wine*, *cultivar*, *Anjou*, *vine*, *fish*, *Loire*, *Terroir* in France, and *South Africa*, *wine*, *white wine*, *grape*, *fish*, *and cultivar* in SA.

Finally, emotional words were more frequent in the South African corpus (6.2%) than in the French one (1.8%). The SA Corpus included three emotional words: *pleasant*, *joy*, *delicious* and the French corpus a single one: *pleasant.*

#### 3.1.2. Prototypical Analysis

The prototypical analysis was carried out on the lemmatized words with a frequency of elicitation greater than two. The four representational zones resulting from this analysis are presented in Table 4 for French consumers and Table 5 for SA consumers. A frequency cut-off point of 10 and an importance cut-off point of five were used for both corpora. 

The French central core is defined by words linked mostly to the wine itself or to its origin (*Wine*, *Cultivar*, *Vine*, *Grape*, *Terroir*, *Loire*). Only two words are linked to consumer’s experience of the wine (*Taste*, *Fruit*). In contrast, the SA central core is clearly linked to wine experience with only sensory words (*Fruity*, *Acidic*, *Crisp*, *White*, *Fresh*, *Dry*).

The differences between the French and SA corpus appear also in the first periphery whose role is to consolidate the central core and allow for some flexibility in the representation. The French first periphery is linked to the mode of consumption of the wines with words such as *aperitif*, *meal*, or *fish*. The SA first periphery span reflects again the salience of the sensory dimension of Chenin blanc wines (*Wood*, *Tropical*) with the addition of a more contextual term (*summer*). For the French corpus sensory (*Fresh*, *Aroma*, *White*, *Yellow*, *Bitter*, *Light*, *Rough* …), experiential (*tasting*, *conviviality*, *friends*, *sun*, *festive*, *summer*) and emotional (*pleasant*) words appear mostly in the second and third periphery indicating that these dimensions are salient for subgroups of consumers only or even idiosyncratic. The inverse is observed in the SA corpus: Origin and Viticulture/Enology words (Wine, White wine, Grape, Variety) appear in the second periphery along with emotional words (pleasant, joy, delicious). It seems thus that the emotional dimension might be more salient than the sensory dimension for a subgroup of SA consumers. The only origin-related word (South Africa) appears only in the third periphery of the SA corpus along with some less consensual sensory words.

#### 3.1.3. Valence Ratings

Valence ratings are shown in the last two columns of Table 3. All word categories were rated positively, and no significant differences were found between countries.

### 3.2. Sorting Task

#### 3.2.1. Sensory Descriptor Analysis

The comparison of the descriptors used in the four conditions shows that the criteria used to sort the wines differ with the consumers’ COO (Table 6). SA participants used mostly sensory words describing the aroma of the wines. Wood was clearly an important criterion for them, along with fruit and acidity. French participants used more frequently evaluative words, such as pleasant and unpleasant or good length or strong.

A specificity analysis was carried out to compare the words used in the two conditions (with and without information) within each country. This amounts to determine, based on a hyper-geometric law, whether a word occurs more frequently in a given condition than in the complete corpus [37]. The data suggest that providing information on the origin of the wines affected French participants more than SA participants. In both conditions, SA participants used sensory descriptors to describe the groups of wines they formed in the sorting task, and the information they received did not appear to change their sorting strategy. In contrast, providing information on the origin of the wines to French participants led to an increased use of the words Loire, South Africa and familiar, suggesting a different sorting strategy more deliberately based on the origin of the wines.

#### 3.2.2. Wine Positioning

**Figure 1:**
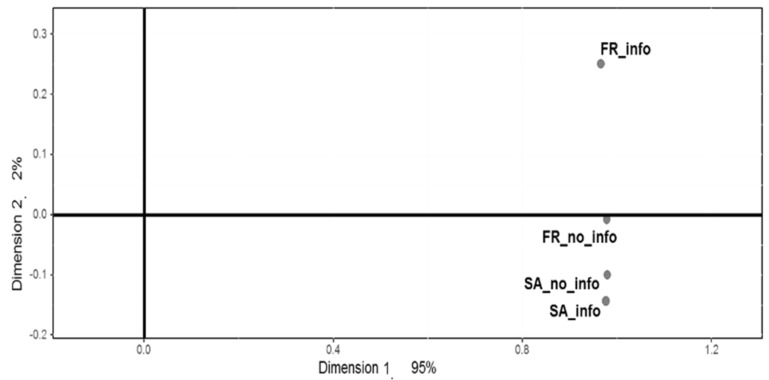
Global Distatis analysis: RV coefficient map.

Figure 1 shows the Distatis RV map comparing the sorts obtained in the four conditions. The proximity between the conditions on the map reflects the similarity of the sorting data obtained in these conditions. The proximity between the two SA conditions indicates that SA participants sorted the wines similarly with and without information. This is attested by the high value of the RV coefficient calculated between these two conditions (RV = 0.63). In other words, providing information on the origin of the wines did not affect SA participants. The distance between the two French conditions is higher than that observed for the two SA conditions (RV = 0.31), indicating that French participants were more affected by the information on the origin of the wines.

**Figure 2:**
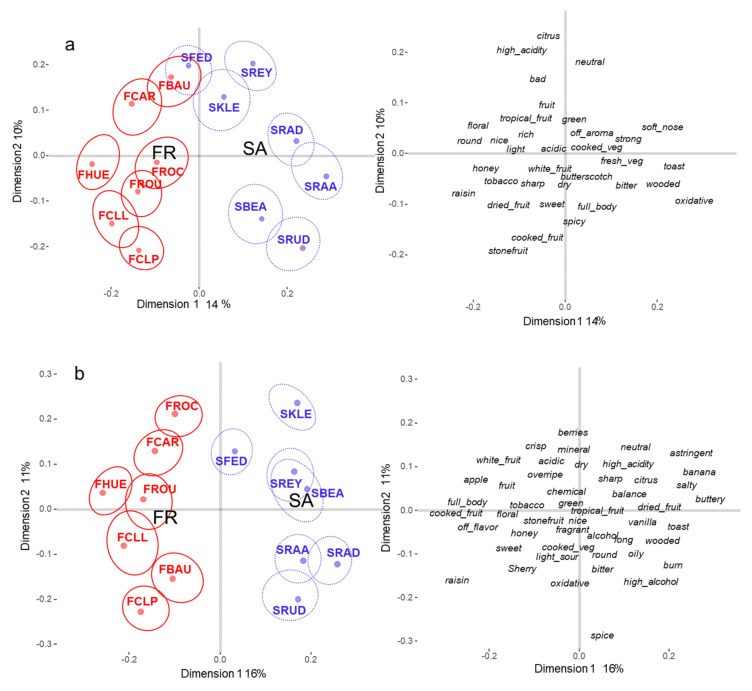
Partial Distatis analyses of the sorting task carried out in South Africa (SA) in the conditions (**a**) without information, and (**b**) with information. The confidence intervals were computed with a bootstrap resampling technique with replacements (95% risk). The French wines are represented in red and the SA wines in blue (See Table 1 for the correspondence between codes and wines). The French (FR) and South African (SA) barycenters and the descriptors were projected as supplementary data on the compromise space.

To have a better understanding of the effect of information on French and SA participants, the analysis was separated by conditions. Figure 2a,b represent the compromise maps obtained from the SA sorting data without and with information, respectively. The structure of the compromise space is quite similar in the SA conditions without and with information. In both conditions, the first dimension clearly opposes the SA and French wines (r = 0.95, *p* < 0.001). A one-way analysis of variance showed a significant effect of wine origin on the projection of the wines on the first dimension. This effect is slightly more important in the with information condition [F(1,12, = 99.76, *p* < 0.0001, R^2^ = 0.90], than in the without information [F(1,12) = 39.43, *p* < 0.0001, R^2^ = 0.77]. The projection of the wine descriptions shows that this first dimension is a sensory dimension opposing woody and vegetal wines (mostly SA wines) to fruity and floral wines (Mostly French wines).

The positions of the wines on the second dimension are also quite similar in the two conditions (*r* = 0.54, *p* < 0.05) with the exception of wine FBAU which projected negatively in the condition with information and positively in the condition without information. In this last condition, the wine FBAU is not significantly different from the wine SFED when these two wines were rather distant in the information condition. The description of this dimension is somewhat different in the two conditions. When no information was provided, the second dimension opposes ripe and rich (*stone fruit*, *cooked fruit*, *spicy full body*, *sweet*, *oxidative*) to high acidity wines (*citrus*, *neutral*, *bad*, *tropical*). With information, it opposes oxidative (spice, raisin, oxidative, sherry) to fresher wines (berry, crisp, neutral white fruit banana, mineral). The wine FBAU was described as bad and high acidity when no information was provided and sherry and oxidative in the information condition.

**Figure 3:**
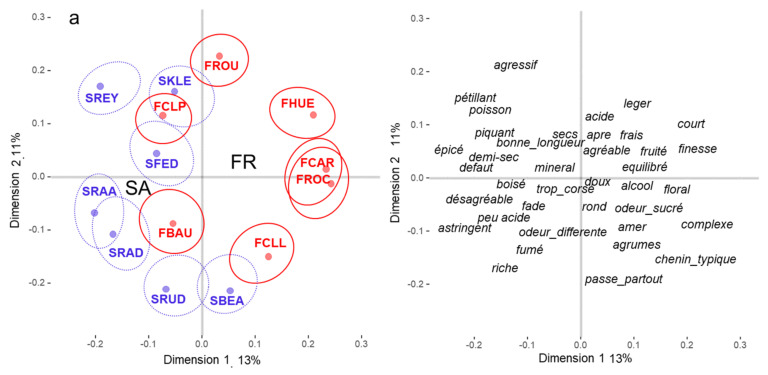

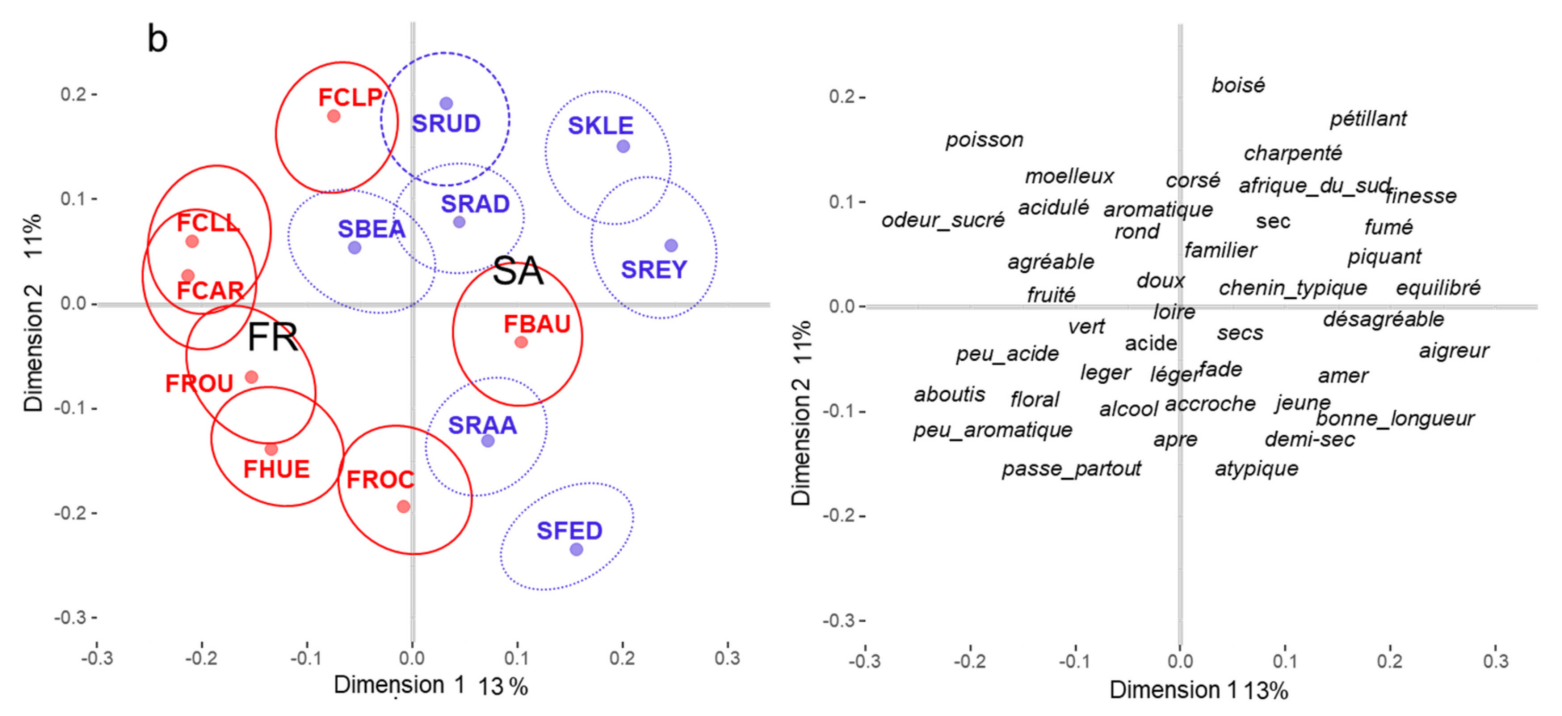
Partial Distatis analyses of the sorting task carried out in France in the conditions (**a**) without information, and (**b**) with information. The confidence intervals were computed with a bootstrap resampling technic with replacements (95% risk). The French wines are represented in red and the SA wines in blue. (See Table 1 for the correspondence between codes and wines). The French (FR) and South African (SA) barycenters and the descriptors were projected as supplementary data on the compromise space. The translation of the French terms can be found in Appendix A.

Figure 3a,b represent the compromise maps obtained from the French sorting data without and with information, respectively. The structure of the French spaces seems to be more affected by the information provided prior to the sorting task than the SA spaces. In both conditions, the first dimension tend to separate again French from South African wines but less clearly than for the South African spaces [F(1,12) = 11.26, *p* < 0.01, R^2^ = 0.48; F(1,12) = 10.99, *p* < 0.01, R^2^ = 0.48 in the with and without information conditions respectively]. The projection of the wine descriptions shows that contrary to what was observed with SA spaces, the first dimension of the French spaces is not dominated by a woody vs. fruity dimension. It seems rather to be a hedonic dimension with French wines being more positively evaluated (*finesse*, *complex*, *typical chenin*, *floral*, *fruity*) than SA wines (*unpleasant*, *little acid*, *astringent*, *fault*, *spicy*), especially in the condition without information on the origin of the wines. The hedonic separation of the wines is less clear in the information condition where both negative terms such as *unpleasant* or sourness and positive terms such as *balanced*, *typical chenin*, finesse, are associated with SA wines suggesting inter-individual differences in the evaluation of these wines.

In the condition without information, the second dimension is relatively close to that observed in the SA conditions with an opposition between aggressive and richer wines. When information on the origin of the wines was provided, French participants associated more woody and full-bodied wines with South African wines (*woody*, *framed*, *full-bodied*, *aromatic*, *South Africa*) and opposed them to lighter wines (*little aromatic*, *average*, *achieved*, *floral*, *light*) mostly French. Interestingly, the wines described as more typical and familiar were SA wines. Finally, as in the SA space, the wine FBAU was described with rather negative terms (*different odor*, *unpleasant*, *sourness*).

## 4. Discussion

The objective of this article was to explore the effect of consumers’ and wines’ COO on Chenin blanc perception in two countries—France and SA—with a very different history of Chenin blanc production and consumption. Whereas France belongs to the so-called “old world” of wine, who classically label wines by appellation or specified regions, SA belongs to the “new world” who tend to rather label wine by grape variety. We were interested in the effect of these historical and experiential differences on the construction of consumers’ Chenin blanc representations and categorization.

The word association task revealed the existence of a structured representation for both groups of consumers, but the wine attributes around which this representation is organized are quite different. SA representations are clearly organized around the experience consumers have of the wines. The terms most important and more frequently cited by SA participants were all linked to sensory aspects of the wines: fruity, wood, acidic, crisp, white; tropical fresh, dry. In line with this result, the saliency of wine taste for South African consumers was previously reported in two studies [38,39]. Both studies used a best-worst scaling (BWS) method to evaluate the importance of 13 attributes on wine purchase for consumers from different generations. The first study [38] report that for Y generation, the highest BWS score was obtained for the attribute “wine tasted previously” followed by the attribute “someone recommended it”. These two attributes were also reported as being the two most important wine attributes for an older group of participants (above 41-year-old) in the second study [39]. Sensory personal experience with the wine seems thus to be the starting point of the construction of wine representation in SA and origin does not seem to play a crucial role in this process.

Region of origin was the second least important attribute in [38], whereas in [39] this attribute obtained a 0 BWS score for the younger group and a score of 0.64 for the older one, showing that the origin of the wine is slightly more important for older participants than younger ones. This attribute is, however, much less important than the taste attribute. In our study, the term “South Africa” was cited by only nine participants and was judged as being not very important. This suggests that there is not a strong link between Chenin blanc and South Africa in SA consumers’ representation. As a comparison, *Fruity* was cited by 34 participants and judged as quite important. The most important term for SA consumers was “delicious”, reinforcing the central role of the personal experience in their representation of Chenin blanc.

Personal experience was much less salient for French participants whose representations were structured around the wine itself, its origin, how it is made and with what it is drunk. The terms Anjou, Loire, and Terroir were among the most frequent and important terms for French participants along with Wine, Grape variety and Vine. Only two terms were related to the experience of the wine: Taste and Fruit. Experiential and emotional terms were important only for subgroups of consumers. The terms linked to the sensory dimension of the wine were very general compared to those used by SA participants. Again, these results can be related to a previous study using the BWS approach in 12 countries [40]. This study showed that while “wine tasted previously” and “someone recommended it” were highly important across most countries, “origin” and “food matching” were more important in France. Likewise, Ginon and coworkers [2] found that, in France, “production region” and “food and wine pairing” were more important than “grape variety” or “previous experiences”. Food matching terms (fish, shellfish, and meals) were also frequently cited in our study, but they were not judged as being very important.

Why do French and SA consumers’ representations of Chenin blanc differ so much? Social representations are a construction of reality by the individuals, and as such, they are deeply rooted in cultural history [41]. Consumers’ environment, participate in the construction of their representations. In the case of wine, consumption practice plays a differentiating role [40]. In other words, the differences we observed between French and SA representations of Chenin blanc reflect differences in cultural history and social practices. France is a traditional wine-producing country with a highly hierarchical appellation system based on wine origin. Previous work emphasized that the wine social representations of French consumers are associated with aspects of national identity such as the traditional cheese-wine association or regional identity [42,43,44]. The French wine industry is present in numerous wine regions, each one with a strong identity. In this context, wine is perceived as a mode of regional differentiation; for example, consumers do not talk about a chardonnay wine, but a chardonnay from burgundy or a chardonnay from the south of France. Likewise, they do not talk about a Chenin blanc but an Anjou blanc or Vouvray, which explains the frequency of occurrence of these terms in the word association task. In contrast, like many new-world wine countries, wine retail in SA is more organized around grape variety and brand than around origin. Wine associations such as the Chenin blanc Association (CBA) have emerged to promote specific wine cultivars, whereas the same type of association is organized regionally in France (e.g., Interloire, interprofession des vins du val de Loire) to promote the wines from specific regions. The promotion of wine cultivars in SA emphasizes the sensory dimension of wine. For example, the CBA proposes a classification of Chenin blanc from SA into six different styles: Fresh and Fruity, Rich and Ripe Wooded, Rich and Ripe Unwooded, Rich and Ripe slightly sweet, Sparkling and Sweet [26]. This association also developed a Chenin blanc aroma wheel. The emphasis on sensory aspects of Chenin blanc may explain the central role of this dimension in SA consumers’ representations.

The difference observed between French and SA consumers’ representation was reflected in the wine sorting task. SA participants used mostly sensory words describing the aroma of the wines to explain the groups of wine they formed, whereas French participants used more frequently evaluative words such as pleasant and unpleasant or good length or strong. Interestingly, the information provided on the origin of the wines led French participants to use more terms related to origin than SA participants who used the same sensory terms in the two conditions.

The sensory-based strategy adopted by SA participants led to a clear separation between SA wines described as rather woody and vegetal wines and French wines described as fruity and floral. This separation between South African and French wines is less clear for French participants. This seems somewhat paradoxical as French participants seemed to have emphasized the origin dimension in the informed condition using words such as Loire or South Africa. This apparent paradox might be explained by the fact that the informed condition might have activated some stereotypes among French participants but that those stereotypes of French consumers do not conform to the sensory reality of French and SA wines. Such a phenomenon was already reported for French and New Zealand Sauvignon Blanc [45]. The explanation put forwards by the authors was that French participants did not have prior exposure to New Zealand Sauvignon wines and thus, their representation of these wines reflected more a consumers’ COO effect: the wines judged the more positively were considered as French. The same might have occurred here, making the separation between French and SA Chenin blanc fuzzier.

Besides providing new insights into cultural differences, our findings have important implications for the marketing and export activities of the wine industry, in particular in a globalized world. For example, sensory marketing seems quite adapted in SA, since SA participant’s representations are mainly organized around a sensory dimension and wine COO does not seem to play a major role.

## 5. Conclusions

Our data align with, but also add significantly to, previously published data on the mental representation of wine in “new” and “old” world wine countries. We have shown that SA and FR consumers have quite different mental representations of dry Chenin blanc wines, the former being more based on their experience of the wine including the sensory, emotional and contextual aspects and the latter built around the idea of origin. Chenin blanc is likely a good model to reveal such fundamental differences in wine appreciation and representation since its sensory profile is generally less well defined than those of other varietals such as Sauvignon blanc or Chardonnay. In South Africa, wine makers are still exploring the wine stylistic potential of the variety, while in France, Chenin blanc is not strongly present as a varietal category. Sensory descriptors therefore are likely to be less influenced by expectations, and may represent a more accurate reflection of the mental representation of wines in general.

It is important to note that the wines evaluated here are a snapshot of Chenin blanc styles. Chenin blanc remains a highly dynamic category. Furthermore, while regionalism in SA is less of a feature than in France, Chenin blanc wines of some regions, such as the Swartland and Breedekloof, have recently achieved critical acclaim as an expression of regionality. Future research will be able to assess whether such trends will impact the mental representation of the product.

## Figures and Tables

**Table 1:** Chenin blanc wines used in the experiment.

Country	Code	Wine	Origin	Vintage	Producer	Price (€)
France	FBAU	Clos du Papillon	Savennières	2010	Florent Baumard	24
France	FCLP	Les Clos Du Papillon	Savennières	2011	Domaine du Closel	22
France	FCLL	La Jalousie	Savennières	2013	Domaine du Closel	14
France	FHUE	Le Haut Lieu	Vouvray	2012	Domaine Huet	12
France	FCAR	Vouvray	Vouvray	2012	Vinent Careme	15
France	FROC	Insolite	Saumur	2011	Domaine des Roches Neuves	17
France	FROU	Les Terasses	Anjou	2012	Chateau de la Roulerie	14
SA	SFED	Hoeksteen	Stellenbosch	2013	The Fledge & Co	4
SA	SRAA	Old vines	Stellenbosch	2012	Raats	9
SA	SREY	Chenin blanc	Stellenbosch	2013	Reyneke	6
SA	SBEA	Hope Marguerite	Botrivier	2013	Beaumont	7
SA	SRAD	Renaissance	Stellenbosch	2013	Radford Dale	4
SA	SRUD	De Tradisie	Stellenbosch	2010	Rudera	8
SA	SKLE	Family Reserve	Stellenbosch	2013	Kleine Zalze	7

**Table 2:** Participants’ demographic information expressed as absolute frequencies.

		SA		France	
		With Information	Without Information	With Information	Without Information
Age	< 40	38	37	25	23
	> 41	15	17	24	27
Gender	F	39	40	25	26
	M	14	14	24	24
Freq consumption	Daily	1	2	.	.
	Week	9	6	5	5
	Month	43	47	43	45
Different Chenin *	< 5	15	9	22	16
	5 to 10	16	22	15	23
	10 to 20	4	15	7	9
	> 20	18	8	2	2

* How many different Chenin blanc wines did you taste in the past year? for each sorting task condition (with and without COO information).

**Table 3:** Frequency and average valences of elicited categories in the word association test using the phrase “Chenin blanc sec” as stimulus.

Categories	SA (*n* = 107)	FR (*n* = 99)	Chi-Square	*p* Value	Valence SA	Valence FR
Sensorial	316	104	107.01	**0.00001**	5.39	5.24
Viti/Enology	66	146	30.19	**0.00001**	6.05	6.08
consumption mode	63	72	0.60	0.43857	6.15	6.78
Emotional	33	9	13.70	**0.00021**	5.74	5.88
Origin	20	76	32.67	**0.00001**	6.00	5.76
Food	17	43	11.27	**0.00079**	6.12	5.56
Other	12	29	7.05	**0.00793**	5.33	4.10
Usage	8	21	5.83	**0.01578**	5.63	5.19
*Total*	*535*	*500*				

**Table 4:** Prototypical analysis of the representation of “Chenin blanc” for the French participants. High importance > 5; Low importance ≤ 5; High frequency > 10; Low frequency ≤ 10.

	High Importance	Low Importance
High frequency	Vin (wine) Cépage (Cultivar) Anjou Vigne (Vine) Raisin (Grape) Gout (Taste) Loire Fruit (Fruit) Terroir	Apéritif (Aperitif) Poisson (Fish) Repas (Meal)
Low frequency	Alcool (Alcohol) Frais (Fresh) Dégustation (Tasting) Savannière Vin blanc (White wine) Arôme (Aroma) Blanc (White) Jaune clair (Pale Yellow) Saumur Agréable (Plaisant) Verre (Glass) Convivialité (Conviviality) Amis (Friends) Soleil (Sun) Amer (Bitter) Sec (dry)	Fruit de mer (Shell fish) Boire (To drink) Couleur (Color) Bouteille (Bottle) Crustacés (crustacean) Léger (Light) Kir Âpre (Rough) Chemin (path) Région (Region) Acide (Acidic) Festif (Festive) Clair (Clear) Famille (family) Été (Summer)

**Table 5:** Prototypical analysis of the representation of “Chenin blanc” for the South African participants. High importance > 5; Low importance ≤ 5; High frequency > 10; Low frequency ≤ 10.

	High Importance	Low Importance
High frequency	Fruity Acidic Crisp White Fresh Dry	Wood Summer Tropical
Low frequency	Wine Cold White_Wine Grape Clear Refresh Honey Smooth Guava Nice Balance Taste Aromatic Grass Ice Variety Light Pleasant Joy Spicy Delicious	South_Africa Yellowish Bitter Green Citrus Mineral Floral Sour Apple Food Butter Astringent Sweet Cooked_Veg Friends Straw Fish Unwooded Green_Apple Wine_Tasting Alcohol Herbaceaous Not_sweet

**Table 6:** Specificity analysis of the words generated during the sorting task in the two conditions (with and without information) within each country. Only words significant at the alpha risk 5% are presented.

	With Information		Without Information	
Country	Word	Frequency	Word	Frequency
FR	Loire	3.3	bonne_longueur (good length)	3.49
	âpre (rough)	3.6	complexe (complex)	1.1
	Afrique_du_sud (SA)	0.9	agrumes (citrus)	0.89
	moelleux (off-dry)	0.8	fumé (smoky)	1.85
	aboutis (achieved)	1.5	frais (fresh)	1.64
	souple (supple)	0.8	riche (rich)	0.68
	familier (familiar)	0.7	défauts (faulty)	1.64
	léger (light)	1.7	astringent (astringent)	1.51
	vert (green)	1.5	piquant (pungent)	4.04
	accroche (grippy)	1.2	chenin_typique (typical chenin)	1.92
	pétillant (sparkling)	1.7	court (short)	1.03
SA	vanilla	2.3	butterscotch	2.33
	stonefruit	3	acidic	7.4
	oily	1.1	light	2.68
	mineral	2.4	rich	1.34
	tobacco	3.5	soft_nose	0.78
	dried_fruit	3.2	fruit	8.32
	crisp	1.3	strong	1.34
	alcohol	1.1	full_body	0.99
	overripe	1.6	off_aroma	3.66
	butternut	0.6	spicy	2.11
	astringent	0.6	sweet	6.06
	long	0.6	sharp	1.69
	pineapple	2.2	bad	0.99
	balance	1		
	more_body	0.5		
	burn	0.5		
	dusty	0.8		
	banana	0.7		

## Appendix A

**Table A1:** English translation of words generated in both condition of the sorting task in France.

aboutis	achieved	épicé	spicy
accroche	rough	équilibré	balanced
acide	acid	fade	bland
acidulé	acidulous	familier	familiar
afrique_du_sud	South Africa	finesse	finesse
agréable	pleasant	floral	floral
agressif	aggressive	frais	fresh
agrumes	citrus	fruité	fruity
aigreur	sourness	fumé	smoke
alcool	alcohol	jeune	young
amer	bitter	léger	light
apre	harsh	Loire	Loire
aromatique	aromatic	mineral	mineral
astringent	astringent	moelleux	soft
atypique	atypical	odeur differente	different odor
boisé	wooded	odeur sucré	sweet smell
bonne_longueur	good_persistence	passe partout	average
charpenté	framed	pétillant	sparkling
chenin_typique	typical chenin	peu acide	little acid
complexe	complex	peu aromatique	little aromatic
corsé	full-bodied	piquant	pungent
court	short	poisson	fish
défaut	fault	riche	rich
demi-sec	off-dry	rond	round
désagréable	unpleasant	sec	dry
doux	soft		

## References

[1] Lacey S., Bruwer J., Li E. (2009). The role of perceived risk in wine purchase decisions in restaurants. Int. J. Wine Bus. Res..

[2] Ginon E., Ares G., Issanchou S., dos Santos Laboissière L.H.E., Deliza R. (2014). Identifying motives underlying wine purchase decisions: Results from an exploratory free listing task with Burgundy wine consumers. Food Res. Int..

[3] Atkin T., Johnson R. (2010). Appellation as an indicator of quality. Int. J. Wine Bus. Res..

[4] Verlegh W., Steenkamp J. (1999). A review and meta-analysis of country-of-origin research. J. Econ. Psychol..

[5] Ashton R.H. (2014). Nothing good ever came from New Jersey: Expectations and the sensory perception of wine. J. Wine Econ..

[6] Veale R., Quester P. (2009). Do consumer expectations match experience? Predicting the influence of price and country of origin on perceptions of product quality. Int. Bus. Rev..

[7] Rodrigues H., Rolaz J., Franco-Luesma E., Sáenz-Navajas M.P., Behrens J., Valentin D., Depetris-Chauvin N. (2020). How the country-of-origin impacts wine traders’ mental representation about wines: A study in a world wine trade fair. Food Res. Int..

[8] Foroudi P., Cuomo M.T., Rossi M., Festa G. (2019). Country-of-origin effect and millennials’ wine preferences—A comparative experiment. Br. Food J..

[9] Nelgen S., Pinilla V., Anderson K. (2017). Global Wine Markets, 1860 to 2016: A Statistical Compendium.

[10] Augustyn O.P.H., Rapp A. (1982). Aroma components of *Vitis vinifera* L. cv. Chenin blanc grapes and their changes during maturation. S. Afr. J. Enol. Vitic..

[11] Mateo J.-J., Jiménez M. (2000). Monoterpenes in grape juice and wines. J. Chromatogr. A.

[12] Tominaga T., Furrer A., Henry R., Dubourdieu D. (1998). Identification of new volatile thiols in the aroma of *Vitis vinifera* L. var. Sauvignon blanc wines. Flavour Fragr. J..

[13] Guichet A., Maury X., Deneulin P., Pages J., Asselin C. (2017). Les Rendez-Vous Du Chenin: Pour Une Analyse de la Diversité Sensorielle des Vins D’expression.

[14] Pages J. (2005). Collection and analysis of perceived product inter-distances using multiple factor analysis: Application to the study of 10 white wines from the Loire Valley. Food Qual. Prefer..

[15] Valente C.A., Bauer F.F., Venter F., Watson B., Nieuwoudt H.H. (2018). Modelling the sensory space of varietal wines: Mining of large, unstructured text data and visualisation of style patterns. Nature Sci. Rep..

[16] Boursiquot J.M., Grondain V. Le Chenin: Origines, Caractéristiques et Variations. Le Chenin: Histoire et Actualités, Territoires du vin.

[17] Rioux D., Courtin V., Cesbron S., Redois F., Assselin C., Girault P. (2017). Les Terres de Chenin: Des terroirs de base représentatives de l’Anjou, du Saumurois et de la Touraine. Le Val de Loire Terres de Chenin p296–303.

[18] Mora J., Mulet A. (1991). Effects of Some Treatments of Grape Juice on the Population and Growth of Yeast Species During Fermentation. Am. J. Enol. Vitic..

[19] Jolly N.P., Augustyn O.P.R., Pretorius I.S. (2003). The use of *Candida pulcherrima* in combination with *Saccharomyces cerevisiae* for the production of Chenin blanc wine. S. Afr. J. Enol. Vitic..

[20] Marais J., Jolly N. (2005). Effect of yeast strain and less contact on Chenin blanc wine quality. Winetech Technical Yearbook.

[21] Reynolds A.G., Edwards C.G., Cliff M.A., Thorngate J.H., Marr J.C. (2001). Evaluation of Yeast Strains during Fermentation of Riesling and Chenin blanc Musts. Am. J. Enol. Vitic..

[22] Aleixandre-Tudo J.L., Weightman C., Panzeri V., Nieuwoudt H.H., Du Toit W.J. (2015). Effect of Skin Contact Before and During Alcoholic Fermentation on the Chemical and Sensory Profile of South African Chenin Blanc White Wines. S. Afr. J.Enol. Vitic..

[23] Botha A., Du Toit W., Brand J., Kidd M., Groenewald N. (2020). The Effect of Different Oak Products Used during Fermentation and Ageing on the Sensory Properties of a White Wine over Time. Foods.

[24] James T. (2013). Wines of the New South Africa.

[25] Loubser F.H. (2008). Chenin Blanc Table Wines in South Africa Cape.

[26] Goode J. (2012). The Different Faces of Chenin Blanc. https://wineanorak.com/2021/06/15/the-different-faces-of-south-african-chenin-blanc/.

[27] SAWIS SA Wine Industry 2017 Statistics NR42. http://www.sawis.co.za/info/download/Book_2017_statistics_year_english_final.pdf.

[28] Gillet F., Assselin C., Girault P. (2017). Approche économique des AOP de Chenin du Val de Loire et des autres productions mondiales. Le Val de Loire Terres de Chenin p296–303.

[29] Flament C., Rouquette M.L. (2003). Anatomie des Idées Ordinaires Comment Etudier Les Représentations Sociales.

[30] Abric J.C., Abric J.C. (2003). La recherche du noyau central et de la zone muette des représentations sociales. Méthodes D’Étude des Représentations Sociales.

[31] Moliner P., Lo Monaco G. (2017). Méthodes D’association Verbale pour les Sciences Humaines et Sociales.

[32] Chollet S., Lelièvre M., Abdi H., Valentin D. (2011). Sort and beer: Everything you wanted to know about the sorting task but did not dare to ask. Food Qual. Prefer..

[33] SAWIS SA Wine Industry 2015 Statistics. http://www.sawis.co.za/info/download/Liquor_Consumption_Patterns_in_South_Africa_2015_eh_v1_final.pdf.

[34] Vergès P. (1992). L’évocation de l’argent: Une méthode pour la définition du noyau central d’une représentation. Bull. Psychol..

[35] Abdi H., Valentin D., Chollet S., Chrea C. (2007). Analyzing assessors and products in sorting task: Distatis, theory and applications. Food Qual. Prefer..

[36] Abdi H., Dunlop J.P., Williams L.J. (2009). How to compute reliability estimates and display confidence and tolerance intervals for pattern classifiers using the Bootstrap and 3-way multidimensional scaling (DISTATIS). NeuroImage.

[37] Lebart L., Piron M., Morineau A. (2006). Statistique Exploratoire Multidimensionnelle. Visualisation et Inférence en Fouille de Données.

[38] Lategan B.W., Pentz C.D., du Preez R. (2017). Importance of wine attributes: A South African Generation Y perspective. Br. Food J..

[39] Pentz C., Forrester A. (2020). The importance of wine attributes in an emerging wine-producing country. South Afr. J. Bus. Manag..

[40] Goodman S. (2009). An international comparison of retail consumer wine choice. Int. J. Wine Bus. Res..

[41] Rateau P., Moliner P., Guimelli C., Abric J.C., Van Lange P.A., Kruglanski A.W., Higgins E.T. (2011). Social representation theory. Handbook of Theories of Social Psychology.

[42] Lo Monaco G., Guimelli C. (2008). Représentations sociales, pratique de consommation et niveau de connaissance: Le cas du Vin. Les Cah. Int. De Psychol. Soc..

[43] Do V.B., Patris B., Valentin D. (2009). Opinions on Wine in a New Consumer Country: A Comparative Study of Vietnam and France. J. Wine Res..

[44] Mouret M., Lo Monaco G., Urdapilleta I., Parr W. (2013). Social representations of wine and culture: A comparison between France and New Zealand. Food Qual. Prefer..

[45] Parr W., Valentin D., Green J., Dacremont C. (2010). Evaluation of French and New Zealand Sauvignon wines by experienced French wine assessors. Food Qual. Prefer..

